# The Community Reinforcement Approach: An Update of the Evidence

**Published:** 2011

**Authors:** Robert J. Meyers, Hendrik G. Roozen, Jane Ellen Smith

**Keywords:** Alcohol use disorders, alcohol and other drug disorders, substance use disorders, treatment, treatment methods, Community Reinforcement Approach (CRA), Adolescent CRA, Community Reinforcement and Family Training

## Abstract

The Community Reinforcement Approach (CRA), originally developed for individuals with alcohol use disorders, has been successfully employed to treat a variety of substance use disorders for more than 35 years. Based on operant conditioning, CRA helps people rearrange their lifestyles so that healthy, drug-free living becomes rewarding and thereby competes with alcohol and drug use. Consequently, practitioners encourage clients to become progressively involved in alternative non-substance-related pleasant social activities, and to work on enhancing the enjoyment they receive within the “community” of their family and job. Additionally, in the past 10–15 years, researchers have obtained scientific evidence for two off-shoots of CRA that are based on the same operant mechanism. The first variant is Adolescent Community Reinforcement Approach (A-CRA), which targets adolescents with substance use problems and their caregivers. The second approach, Community Reinforcement and Family Training (CRAFT), works through family members to engage treatment-refusing individuals into treatment. An overview of these treatments and their scientific backing is presented.

The Community Reinforcement Approach (CRA) is a comprehensive behavioral treatment package that focuses on the management of substance-related behaviors and other disrupted life areas. The goal of CRA is to help people discover and adopt a pleasurable and healthy lifestyle that is more rewarding than a lifestyle filled with using alcohol or drugs. Multiple research reviews and meta-analyses of the treatment-outcome literature have shown CRA to be among the most strongly supported treatment methods (Finney and Monahan 1996; Holder et al. 1991; Miller et al. 1995, 2003). This article briefly discusses the science behind CRA, and provides an overview of the treatment program. In addition, it discusses two novel variants built upon the CRA foundation. These interventions include an adolescent version of CRA called Adolescent Community Reinforcement Approach (A-CRA), and a program called Community Reinforcement and Family Training (CRAFT), which is designed to engage treatment-refusing substance-abusing individuals into treatment by working through family members.

## Development and Effectiveness of CRA

The most influential behaviorist of all times, B. F. Skinner, largely considered punishment to be an ineffective method for modifying human behavior (Skinner 1974). Thus it was no surprise that, many years later, research discovered that substance use disorder treatments based on confrontation were largely ineffective in decreasing the use of alcohol and other substances (Miller and Wilbourne 2002, Miller et al. 1998). Nate Azrin already was convinced of this back in the early 1970s, when he designed an innovative treatment for alcohol problems: the Community Reinforcement Approach (CRA). Azrin believed that it was necessary to alter the environment in which people with alcohol problems live so that they received strong reinforcement for sober behavior from their community, including family, work, and friends. As part of this strategy, the program emphasizes helping clients discover new, enjoyable activities that do not revolve around alcohol, and teaching them the skills necessary for participating in those activities (see sidebar for a description of CRA procedures).

Research has since supported the premise behind CRA. Studies show that people with substance use disorders report that they are less engaged in pleasant activities compared with healthy controls (Roozen et al. 2008; Van Etten et al. 1998). And other studies found that enriching people’s environment with non–substance-related rewarding alternatives encourages them to reduce their substance use (Correia et al. 2005; Vuchinich and Tucker 1996). Even modern day neurobiology has confirmed that components of addiction treatment should focus on increasing patients’ involvement with alternative reinforcers (Volkow et al. 2003).

In terms of testing CRA itself, studies suggest that it is highly effective. Azrin’s first two studies of the program tested its effectiveness among alcohol-dependent inpatients (Azrin 1976; Hunt and Azrin 1973). The results showed that the new CRA program was more effective in reducing drinking than was the hospital’s Alcoholics Anonymous program. Furthermore, the CRA participants had better outcomes with regard to their jobs and family relationships. Azrin then modified the program slightly to test it with outpatients at a rural alcohol treatment agency (Azrin et al. 1982). He and his colleagues, again, found CRA to be superior to the comparison condition.

A larger outcome study conducted in the 1990s had mixed results, though it did show a benefit of CRA on the immediate outcome. (Miller et al. 2001). For this study, participants had to score in the symptomatic range on two of four measures, including the Addiction Severity Index and the Alcohol Use Inventory. The final sample consisted of people who met an average of 7 of the 9 criteria for alcohol dependence syndrome as defined by the *Diagnostic and Statistical Manual of Mental Disorders, Third Edition, Revised* (DSM–III–R) (American Psychiatric Association 1980). The study compared CRA with a “traditional” treatment. However, because this comparison treatment used a CRA procedure as part of its protocol—teaching one of the participants’ loved ones positive communication skills so he or she could administer disulfiram (Antabuse^®^) in a supportive and caring way— the overlap could have obscured the results somewhat. Another confounding factor may have been that the traditional treatment group included more participants who agreed to take disulfiram in the first place (Miller et al. 2001).

In a study that delivered CRA in a group format to severely alcohol-dependent homeless individuals in a day treatment program, CRA produced significantly greater substance use outcomes than did the standard treatment at the homeless shelter (Smith et al. 1998). Finally, another study discovered that people with antisocial personality disorder could, in fact, respond successfully to a CRA program, even if it highlighted the relationship counseling aspect of CRA (Kalman et al. 2000).

The table provides an overview of Community Reinforcement studies. The first section highlights the trials in which researchers tested “pure” CRA, without any additional programs. Several comprehensive reviews and meta-analyses support the conclusion that CRA is highly effective compared with other alcohol treatments (Finney and Monahan 1996; Holder et al. 1991; Miller et al. 1995, 1998, 2003, 2005; Roozen et al. 2004). Although it is not readily apparent from the table, CRA has been clinically effective for people with varying degrees of alcohol problems and with psychiatric comorbidity, in both rural and urban environments, and for people with goals of either abstinence or reduced use. It also has been modified to expand its reach to people with illicit drug problems, to adolescents, and to people resistant to entering treatment, as will be explained in the following sections.

## CRA plus Contingency Management

Higgins, a researcher who was very interested in using CRA to treat cocaine-dependent individuals, believed that people with cocaine-dependence needed tangible incentives to combat strong urges early in recovery. Thus, he developed a contingency management program to supplement CRA for his work with these patients. The program provided vouchers to participants who submitted drug-free urine samples. In turn, they could exchange the vouchers for goods, such as dinners. A number of early studies demonstrated that CRA plus vouchers outperformed standard treatment programs (e.g., Higgins et al. 1991, 1993, 1994). Another study showed that CRA plus vouchers was significantly better than vouchers alone in terms of improved treatment retention and employment rates, and reduced cocaine use—at least during the treatment phase (Higgins et al. 2003). The CRA plus vouchers program has been used successfully with other illicit drugs as well. For example, people receiving opioid detoxification with buprenorphine had significantly better treatment outcomes if they also received CRA plus vouchers (Bickel et al. 1997). In addition, a recent study with adults who used cannabis determined that long-term outcomes favored clients who received CRA in addition to vouchers as opposed to just vouchers alone (Budney et al. 2006). Thus, the CRA plus contingency management package appears to be a highly successful program for treating individuals who abuse illicit drugs (Bickel et al. 2008; Garcia-Rodriquez et al. 2009).

## The Adolescent Version of CRA: A-CRA

The high rate of illicit substance use among adolescents has been viewed as one of the primary public health problems facing the United States for some time now (Johnston et al. 2001). According to one report, during a relatively recent six-year period (1992–1998), the number of 12- to 17-year-olds who were admitted to public substance use treatment agencies increased by 54 percent (Dennis et al. 2003). Consequently, it is more important than ever to identify *effective* substance use disorder treatment programs for adolescents. A-CRA is a scientifically-based behavioral intervention that is a slightly modified version of the adult CRA program (for descriptions with examples see Godley et al. 2001, 2009).

To begin with, developers of A-CRA modified several of the CRA procedures, and the forms that accompany them, to make them more developmentally appropriate for adolescents. For example, the adolescent versions of the Happiness Scale and the Goals of Counseling form contain additional categories focused on school and friends (Forehand and Wierson 1993). In addition, developers simplified the communication skills training procedure and added an anger management procedure to assist with impulsive, acting-out behavior (Weisz and Hawley 2002).

The main unique element in A-CRA is that it involves caregivers—namely, parents or other individuals who are ultimately responsible for the adolescent and with whom the adolescent is living—in the treatment program. These caregivers attend four sessions: two devoted to the caregiver(s) alone and two set up for the caregiver and the adolescent together. Among other things, the caregiver-alone sessions emphasize parenting “rules.” This is especially relevant because parental rule-setting has been inversely associated with adolescents’ alcohol use over time, and even moderates the presence of a genetic predisposition toward alcohol use (Van der Zwaluw et al. 2009). The program also teaches caregivers several of the basic skills, including communication and problem-solving, that their adolescent has learned in individual sessions. During the sessions with both the adolescent and the caregiver, the therapist guides family members in using positive communication skills with each other as they address problems in their relationship. The group negotiates goals geared toward increasing happiness in the adolescent–caregiver relationship, and adolescents and caregivers practice problem-solving exercises that they are asked to continue outside of therapy.

**CRA Procedures**

**Figure 1:**
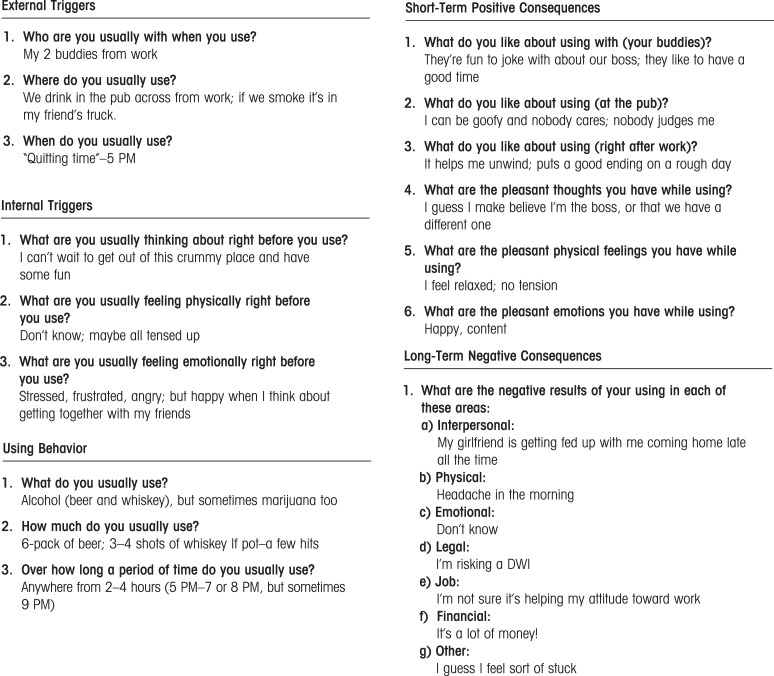
Functional Analysis for Using Behaviors SOURCE: Meyers, R.J., and Smith, J.E. *Clinical Guide to Alcohol Treatment: The Community Reinforcement Approach,* New York: Guilford Press, 1995, pp. 34–35. Adapted with permission.

**Figure 2:**
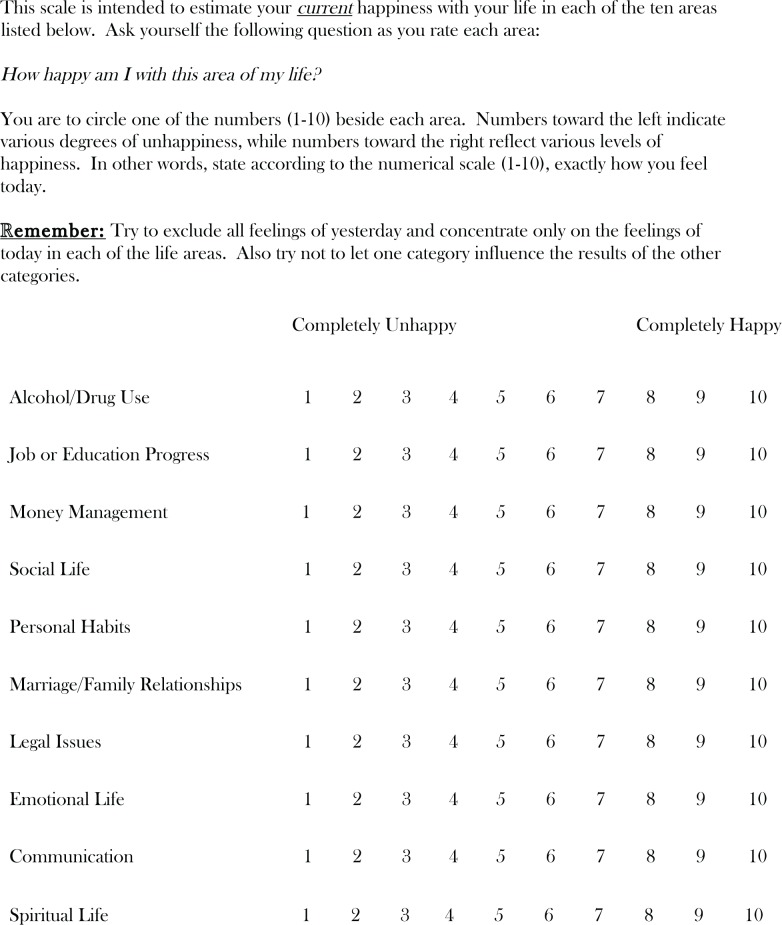
Happiness Scale. SOURCE: Meyers, R.J., and Smith, J.E. *Clinical Guide to Alcohol Treatment: The Community Reinforcement Approach*. New York: Guilfor Press, p.95. Adapted with permission.

The basic CRA procedures include:

(1) *Functional Analysis of Substance Use* explores the antecedents and positive and negative consequences of a client’s substance use. This allows clinicians to identify new behaviors that will be reinforcing to the client while also discouraging alcohol and drug use. A sample of a completed Functional Analysis for a common drinking episode is provided in figure 1. In this example, the client drinks daily after work because it relieves his stress and he enjoys being around people who can empathize and laugh with him about his unpleasant work situation (Short-Term Positive Consequences). It would therefore be critical to help this client find ways to receive empathy, have fun, and alleviate stress without drinking. It also would be important to explore precisely what it is about his job/boss that is so stressful, and then address that directly through communication skills training, problem solving, or entertaining the notion of a different job or a transfer. Because he reported concern over his girl-friend’s feelings about his drinking (Long-Term Negative Consequences), it would be important to see what type of role she might play in satisfying these objectives. Importantly, the client understands at some level that drinking excessively with these friends every night is not necessarily resolving his work problem. Consequently he might be willing to “sample” some small changes in his daily pattern to see how they feel. And because his weekday social network revolves entirely around drinking, considerable time would be devoted to using CRA’s Social/Recreational Counseling (see procedure 6, below).
(2) *Sobriety Sampling* is based on the belief that it can be counterproductive for therapists to tell clients that they can never drink again for the rest of their lives (even if the client should not). Sobriety Sampling is a gentle movement toward long-term abstinence that begins with a client’s agreement to sample a time-limited period of abstinence. The client and therapist negotiate the period of time, and the therapist then helps the client develop a plan and the tools for achieving this goal.
(3) *CRA Treatment Plan* begins with the Happiness Scale (figure 2) to let clients know that all aspects of their lives are important, not just their substance using behavior. It also provides the structure for easily identifying areas of discontent and later signs of progress. Clients select areas from the Happiness Scale to work on, and then use the Goals of Counseling form to establish meaningful, objective goals in these areas, and highly specified methods for obtaining them.
(4) *Behavioral Skills Training* uses instruction and role-plays with feedback to teach three basic skills: (a) problem-solving, which breaks overwhelming problems into smaller ones while offering a step-by-step framework for addressing them, (b) communication skills, which teaches a positive interaction style that involves simple constructs such as offering to help and verbalizing empathy, and (c) drink/drug refusal training, which helps identify high-risk situations and then teaches assertiveness.
(5) *Job Skills Training* provides basic steps for obtaining and keeping a valued job. Having a meaningful job generally is considered a significant source of alternative reinforcement that is incompatible with problematic substance use (see Azrin and Besalel 1980 for the Job Club Counselor’s Manual).
(6) *Social and Recreational Counseling* helps clients discover that they can enjoy life without drugs and alcohol by providing them with opportunities to sample new social and recreational activities. In referring to the case of the man who drank at the pub after work each day with his buddies, it should be readily apparent that helping him develop a new satisfying social life would be critical for sustained abstinence. Although he was drinking for other reasons as well, the outlet to laugh with friends after a hard day at work was highly reinforcing to him. CRA therapists would help him find a highly reinforcing alternative way to satisfy that need, as opposed to simply encouraging him to find a substitute activity.
(7) *Relapse Prevention* teaches clients how to identify high-risk situations and to anticipate and cope with a relapse. Patients practice various behavioral skills as part of this procedure, including drink/drug refusal training and problem solving, and may learn several specific relapse prevention techniques, such as (a) the early warning monitoring system, which involves enlisting the support of someone to help watch for early signs of an impending relapse, and (b) CRA Functional Analysis of Relapse, which is a functional analysis that focuses specifically on a recent relapse.
(8) *Relationship Counseling* focuses on the improving the interaction between the client and his or her partner. CRA programs use a couple’s version of the Happiness Scale along with the Goals of Counseling form, and each member of the dyad requests a minor change from their partner. The couple practices communication and problem-solving skills during this process. Finally, therapists introduce the Daily Reminder to Be Nice as a means for steadily incorporating some of the “pleasantries” back into the relationship, which likely have disappeared (see Meyers and Smith 1995, pp. 171, 174–6, 179 for each of the forms mentioned).

—*Robert J. Meyers, Hendrik G. Rozen, and Jane Ellen Smith*

A national study with 600 participants tested the efficacy of A-CRA, comparing the program with several other treatments, including Motivational Enhancement Therapy/Cognitive Behavior Therapy (with two different lengths of treatment), Multidimensional Family Therapy, and Family Support Network (Dennis et al. 2004). The participating adolescents often had multiple substance use disorders, and approximately 70 percent had symptoms of co-occurring psychiatric disorders. Although a number of the treatments were equally effective statistically, A-CRA was the most *cost*-effective intervention. More recently, the effectiveness of A-CRA was confirmed in a study with homeless youth (Slesnick et al. 2007).

## Community Reinforcement and Family Training (CRAFT)

A sizeable group of individuals with substance use disorders refuse to engage in treatment (Stinson et al. 2005; Substance Abuse and Mental Health Services Administration 2009). Even for those who *do* seek treatment, it may take them 6–10 years after the initiation of drug use (Joe et al. 1999; Wang et al. 2005). This reticence to seek treatment can have tangible consequences. Concerned family members often experience profound emotional and relationship damage from living with a person with an untreated substance use disorder (Kahler et al. 2003; Kirby et al. 2005). Substance use disorders often are associated with intimate partner violence (Fals-Stewart and Kennedy 2005).

CRAFT was designed to address this problem by targeting people who refuse to seek treatment for substance-abuse problems. Derived from the operant-based fundamentals of CRA, CRAFT decidedly does *not* pressure these individuals to attend treatment. Instead, it operates indirectly and gently through a concerned family member, called the Concerned Significant Other (CSO) in the program. CRAFT therapists show CSOs how to change the home environment of the treatment-resistant individual to reward behaviors that promote sobriety and withhold rewards when the individual is using drugs or alcohol (Smith and Meyers 2004; Smith et al. 2008).

For example, assume a husband thoroughly enjoys having his wife (the CSO) join him in some after-dinner activity, such as watching television or playing cards, and that this routinely occurs after the husband has been drinking. After discussing the potential for domestic violence and teaching positive communication skills, a therapist might coach the CSO to have some variation of the following conversation with her husband at breakfast: “I wanted to let you know that I really enjoy sitting and watching our favorite shows together in the evening, but I only will do it from now on when you haven’t been drinking. I want to do everything I can to support your sobriety.” The message would be modified to suit the particular situation, and in some cases the CSO might elect to not even communicate with the substance user about the plan in advance. Regardless, it is critical that the CSO, in this case the wife, follow through with the plan to *only* join her husband if he was sober, and to get up and excuse herself—again, using positive communication skills—if he started to drink.

Learning how to appropriately reward clean/sober behavior is only one aspect of CRAFT, but over time it can become a powerful tool. Importantly, it must be used consistently and applied across a number of different behaviors. Relying upon positive communication throughout the process is critical for success. Furthermore, the appropriate use of this procedure requires that CSOs learn the difference between the reinforcement of clean/sober behavior and enabling. The latter is the CSO’s inadvertent reinforcement of drinking or drug using (Meyers and Smith 1997). Two CRAFT books—a therapist manual (Smith and Meyers 2004) and a self-help book (Meyers and Wolfe 2004)—outline the differences between appropriate reinforcement and enabling, as well as provide comprehensive descriptions of the other CRAFT procedures.

Along with helping to encourage substance abusers to seek treatment, CRAFT also focuses on enhancing the happiness of the CSO overall. Therefore, some of its procedures help CSOs identify the areas of their lives in which they would like to make changes, and then assist in developing strategies to accomplish their goals. For example, assume a mother (CSO) has delayed finishing up her degree at the local college because she has been preoccupied with caring for her substance-abusing 19-year-old daughter. If the CSO noted on her Happiness Scale that she was very unhappy in the job/education category, the therapist would explore whether she wanted to set some goals in that area. A reasonable goal might be to take one college course that semester, and the strategy would involve several steps, including finding out which courses she needed to graduate, which courses were offered at a convenient time, and determining her financial aid status. She would also identify and address obstacles. For example, she might be reluctant to leave for class on evenings when her daughter is high. Acceptable solutions could vary widely, but might involve asking a neighbor to check on the daughter in her absence, or dropping the daughter at a safe location for the evening. A therapist would check progress toward the CSO’s goals weekly, and help modify them as needed.

Studies (see table) have consistently demonstrated that CRAFT is 2–3 times more successful at engaging treatment-resistant individuals in substance abuse treatment than the traditional Al-Anon model and the Johnson Intervention (Johnson 1986). More specifically, studies show that CRAFT successfully engaged approximately two-thirds of the treatment-refusing individuals into treatment, regardless of whether they used alcohol or other drugs problematically (Kirby et al. 1999; Meyers et al. 1999, 2002; Miller et al. 1999; Roozen et al. 2010; Sisson and Azrin 1986). Furthermore, CRAFT worked across ethnicities and various types of relationships, including spouse–spouse, parent–child and sibling–sibling. Generally, substance users engaged in treatment after only 4–6 CSO sessions. Irrespective of whether the substance user engaged in treatment, the CSOs reported a sizeable reduction in their own physical symptoms, depression, anger and anxiety (Dutcher et al. 2009; Kirby et al. 1999; Meyers et al. and 1999, 2002; Miller et al. 1999; Sisson and Azrin 1986). CRAFT demonstrated similar success rates when used with the parents of treatment-resistant adolescents (Waldron et al. 2007).

## Why Therapists Like CRA, A-CRA, and CRAFT

Therapists being trained in CRA, A-CRA, or CRAFT typically report being pleasantly surprised that the treatments and the manuals have flexibility built into them as far as the sequencing, spacing, number, and format for delivering treatment sessions. Therapists appreciate being allowed to retain some autonomy; they recognize that their own clinical skills are relied upon to make certain treatment decisions when it comes to tailoring the menu-driven approach to clients’ individual needs. For example, assume a client does not appear prepared to directly address her substance use in the first session, but she is eager to get a job and she agrees that her social life could use some attention. The CRA (or A-CRA) therapist may choose to address either of these areas first, because both areas will indirectly target the client’s substance use problem also: thus, discussions about obtaining a job might easily bring up mandatory urine tests, and talking about enhancing her social life might introduce the idea of substance-free activities and friends. Therapists also respond favorably to the basic premise of community reinforcement treatments—namely, that the emphasis should be on using reinforcement to affect behavior change. At the same time, therapists are relieved to learn that despite being a non-confrontational treatment, CRA/A-CRA/CRAFT therapists are directive, have clear expectations, and set limits as needed (Meyers and Smith 1995; Smith and Meyers 2004).

## Future Directions

Because the scientific evidence has established that community reinforcement treatments are effective, current lines of research have focused on determining state-of-the-art methods for training therapists (Garner et al. 2009*a*) and for ascertaining which specific procedures in these comprehensive treatment packages are most crucial (Garner et el. 2009*b*). In terms of clinical advances, these treatments are being adopted in various countries around the world, as evidenced by translations of the CRA book into German, Dutch, and Finnish, and the CRAFT book into German, Finnish, and Korean. In addition, clinicians are considering applying CRA and CRAFT to other diagnoses, such as eating disorders (Gianini et al. 2009), and investigating the use of A-CRA for adolescents with comorbid conditions.

## Figures and Tables

**Table:** CRA, A-CRA, and CRAFT Studies

**Year**	**First author**	**Type of substance**	***N***	**Population**	**Control group**	**Setting**	**Exp. intervention**	**Outcome**
**CRA**								
1973	Hunt	Alcohol	16	Adults	Yes	inpatient	CRA	+
1976	Azrin	Alcohol	18	Adults	Yes	inpatient	CRA	+
1982	Azrin	Alcohol	43	Adults	Yes	outpatient	CRA	+
1998	Smith	Alcohol	106	Adults	Yes	outpatient	CRA	+
1994	Azrin	Drugs	26	Youth	Yes	outpatient	CRA	+
1998	Abbott	Opioids	166	Adults	Yes	outpatient	CRA	+
2000	Schottenfeld	Opioids & Cocaine	117	Adults	Yes	outpatient	CRA	=
2000	Kalman	Alcohol	149	Adults	Yes	outpatient	CRA	=
2001	Miller	Alcohol	237	Adults	Yes	outpatient	CRA	+
2001	Azrin	Drugs	56	Youth	Yes	outpatient	CRA	+
2003	Roozen	Opioids	24	Adults	No	outpatient	CRA	NA
2006	Roozen	Tobacco	25	Adults	Yes	outpatient	CRA	=
2007	De Jong	Opioids	272	Adults	No	outpatient	CRA	NA
**CRA and Vouchers**								
1991	Budney	Cocaine	2	Adults	No	outpatient	CRA & Vouchers	NA
1991	Higgins	Cocaine	25	Adults	Yes	outpatient	CRA & Vouchers	+
1993	Higgins	Cocaine	38	Adults	Yes	outpatient	CRA & Vouchers	+
1994	Higgins	Cocaine	40	Adults	Yes	outpatient	CRA & Vouchers	NA
1997	Bickel	Opioids	39	Adults	Yes	outpatient	CRA & Vouchers	+
2003	Higgins	Cocaine	100	Adults	Yes	outpatient	CRA & Vouchers	+
2008	Secades-Villa	Cocaine	43	Adults	Yes	outpatient	CRA & Vouchers	+
2008	Bickel	Opioids	135	Adults	Yes	outpatient	CRA & Vouchers	+
2008	DeFuentes-Merillas	Opioids & Cocaine	66	Adults	Yes	outpatient	CRA & Vouchers	+
2009	Garcia-Rodriquez	Cocaine	96	Adults	Yes	outpatient	CRA & Vouchers	+
**A-CRA**								
2002	Godley	Alcohol & Drugs	114	Youth	Yes	outpatient	A-CRA	+
2004	Dennis	Drugs	300	Youth	Yes	outpatient	A-CRA	=
2007	Slesnick	Alcohol & Drugs	180	Youth	Yes	outpatient	A-CRA	+
**CRAFT**								
1986	Sisson	Alcohol	12	Adults	Yes	outpatient	CRAFT	+
1999	Meyers	Drugs	62	Adults	No	outpatient	CRAFT	NA
1999	Miller	Alcohol	130	Adults	Yes	outpatient	CRAFT	+
1999	Kirby	Drugs	32	Adults	Yes	outpatient	CRAFT	+
2002	Meyers	Drugs	90	Adults	Yes	outpatient	CRAFT	+
2007	Waldron	Drugs	42	Adolescents	No	outpatient	CRAFT	NA
2009	Dutcher	Alcohol	99	Adults	No	outpatient	CRAFT	NA

NOTE: The studies included are considered unique published studies and are available in electronic databases such as PubMed and PsychInfo. The effects of each study are appraised as +, statistically significant effect in favor of the experimental condition; =, no statistically significant difference detected; and NA, Not Applicable.
